# Long-Term Effects on QT Prolongation of Pretomanid Alone and in Combinations in Patients with Tuberculosis

**DOI:** 10.1128/AAC.00445-19

**Published:** 2019-09-23

**Authors:** Hanbin Li, David H. Salinger, Daniel Everitt, Mengchun Li, Angelo Del Parigi, Carl Mendel, Jerry R. Nedelman

**Affiliations:** a Certara, Inc., Princeton, New Jersey, USA; b TB Alliance, New York, New York, USA

**Keywords:** *Mycobacterium tuberculosis*, antimicrobial agents, multidrug resistance, tuberculosis

## Abstract

Concentration-QTc modeling was applied to pretomanid, a new nitroimidazooxazine antituberculosis drug. Data came from eight phase 2 and phase 3 studies. Besides pretomanid alone, various combinations with bedaquiline, linezolid, moxifloxacin, and pyrazinamide were considered; special attention was given to the bedaquiline-pretomanid-linezolid (BPaL) regimen that has demonstrated efficacy in the Nix-TB study in subjects with extensively drug-resistant or treatment-intolerant or nonresponsive multidrug-resistant tuberculosis. Three heart rate corrections to QT were considered: Fridericia’s QTcF, Bazett’s QTcB, and a population-specific correction, QTcN. QTc increased with the plasma concentrations of pretomanid, bedaquiline’s M2 metabolite, and moxifloxacin in a manner described by a linear model in which the three slope coefficients were constant across studies, visits within study, and times postdose within visit but where the intercept varied across those dimensions. The intercepts tended to increase on treatment to a plateau after several weeks, a pattern termed the secular trend. The slope terms were similar for the three QTc corrections, but the secular trends differed, suggesting that at least some of the secular trend was due to the elevated heart rates of tuberculosis patients decreasing to normal levels on treatment. For pretomanid 200 mg once a day (QD) alone, a typical steady-state maximum concentration of drug in plasma (*C*_max_) resulted in a mean change from baseline of QTcN of 9.1 ms, with an upper 90% confidence interval (CI) limit of 10.2 ms. For the BPaL regimen, due to the additional impact of the bedaquiline M2 metabolite, the corresponding values were 13.6 ms and 15.0 ms. The contribution to these values from the secular trend was 4.0 ms.

## Abstract (precis)

Concentration-QTc modeling was applied to pretomanid, a new nitroimidazooxazine antituberculosis drug. Data came from eight phase 2 and phase 3 studies.

## INTRODUCTION

Tuberculosis (TB) is the one of the top 10 causes of death and the world’s leading cause of death from a single infectious disease (1).

Drug therapy for TB relies on combinations of drugs to increase bactericidal activity from different mechanisms of action and to prevent the development of resistance (2). First-line treatment for TB is a combination of the four drugs rifampin, isoniazid, pyrazinamide, and ethambutol. TB with no resistance to these four drugs is called drug susceptible (DS). In 2017, approximately 450,00 people worldwide developed TB that was resistant to rifampin and isoniazid, a condition known as multidrug-resistant (MDR) TB (1). Therefore, the need for innovative antituberculosis regimens is great.

Pretomanid, a new chemical entity of the nitroimidazooxazine class, is under investigation for the treatment of TB. A four-drug regimen of pretomanid with bedaquiline, moxifloxacin, and pyrazinamide has shown promise in a phase 2b study in MDR-TB patients (3) and is currently being tested in both DS- and MDR-TB patients in a phase 2c trial (ClinicalTrials.gov Identifier NCT03338621).

Among the most difficult-to-treat Mycobacterium tuberculosis strains are those causing extensively drug-resistant tuberculosis (XDR-TB), defined as MDR-TB with additional resistance to at least one each of second-line fluoroquinolones and injectable agents (amikacin, capreomycin, or kanamycin). In the phase 3 Nix-TB study (4), the three-drug combination of bedaquiline, pretomanid, and linezolid (BPaL) has demonstrated efficacy in patients with XDR-TB or treatment-intolerant or nonresponsive (TI/NR) MDR-TB. In the BPaL regimen, the dose of pretomanid was 200 mg once a day (QD).

Prolongation of the cardiac QT interval, which may be associated with fatal arrhythmias, is a recognized risk of some marketed drugs (5), including bedaquiline (6) and moxifloxacin (7). The potential for pretomanid to affect the QTc, i.e., the QT interval corrected for heart rate (HR), after single-dose administration was assessed in a thorough QT (TQT)/QTc study (8). The maximum, least-squares-mean, placebo-corrected, change-from-baseline, individual-corrected QT (ΔΔQTcI) value for the 400-mg dose of pretomanid administered alone was 2.7 ms, and for the 1,000-mg dose, it was 4.4 ms (ClinicalTrials.gov Identifier NCT01674218) (unpublished data).

Valuable information on the exposure-response relationship of a drug’s QT-related risk can also be obtained through pharmacokinetic/pharmacodynamic (PK/PD) modeling of data from clinical studies other than the TQT/QTc study that collected plasma drug concentrations concurrently with electrocardiograms (ECGs) (9). Such data were available for pretomanid from six phase 2 and two phase 3 studies in TB patients. Treatment durations in these studies ranged from 2 weeks to 6 months and thus allowed assessment of concentration/QTc behavior during long-term treatment. The analysis reported here uses those clinical data to construct a quantitative model describing the relationship between pretomanid plasma concentrations and the change from baseline in QTc during treatment of up to 6 months’ duration. In addition to evaluation of the QT impact of pretomanid alone, a focus of this report is the QT impact of the BPaL regimen from the Nix-TB study.

## RESULTS

### Data and exploratory analyses.

**(i) Summary of the analysis data set.** An overview of the analysis data set is provided in Tables S1 and S2 in the supplemental material. There were 4,652 posttreatment ECG observations with valid concurrent PK observations from 830 subjects in 26 cohorts treated with a single drug or combinations of two, three, or four drugs. Partner drugs in combinations considered here were bedaquiline, linezolid, moxifloxacin, and pyrazinamide. Most of the subjects had DS-TB and normal QTcF values at baseline. The median HR at baseline was 88.5 bpm.

**(ii) QT and heart rate.** It is known that the QT interval varies inversely with HR, so it is customary to analyze a “corrected” QT interval, QTc, that is adjusted for HR (10). The general form for such a correction is QTc = QT × (HR/60)*^r^*, with HR in beats per minute (bpm). The exponent *r* is chosen empirically such that QTc is no longer correlated with HR. Different correction methods use different values of *r*: Fridericia’s (11) QTcF uses an *r* of 0.33, and Bazett’s (12) QTcB uses an *r* of 0.5.

**FIG 1:**
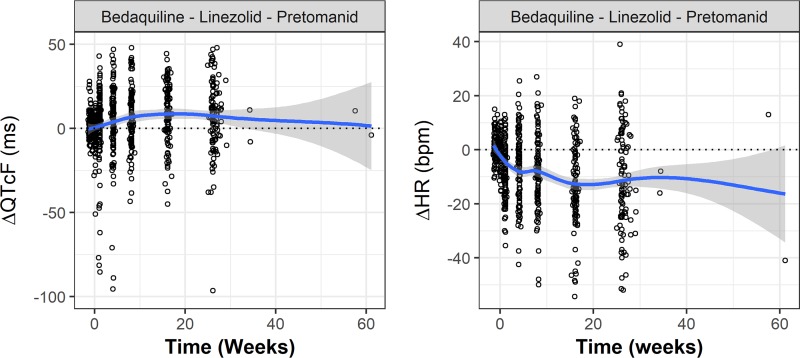
QTcF and heart rate change from baseline versus time in the Nix-TB study. Circles represent observed individual results, a blue line represents statistical smoothing of the data, and the gray area represents 95% confidence intervals of the smooth.

Patients with TB tend to have elevated HRs, which TB treatment tends to normalize (13, 14). The time courses of the change in QTcF and HR from baseline (ΔQTcF and ΔHR) in the Nix-TB study are illustrated in Fig. 1 All subjects in the Nix-TB study were treated with BPaL. ΔQTcF tended to increase after treatment, while ΔHR tended to decrease. Similar trends were also observed in most of the cohorts treated with pretomanid alone or in combination with other drugs. Such trends raise the concern that QTcF may not adequately correct for HR and that therefore the observed increase in ΔQTcF may not accurately reflect the risk of arrhythmia. So, the appropriateness of the correction was investigated using pretreatment ECG data.

**FIG 2:**
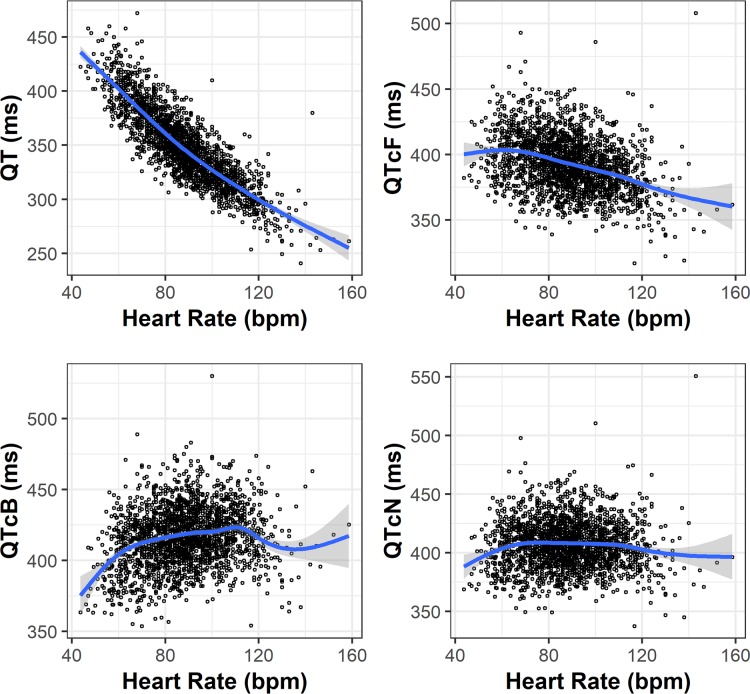
Relationship of pretreatment QT and QTc versus heart rate at pretreatment. QT correction: *r* is 0.33 for QTcF, 0.50 for QTcB, and 0.42 for QTcN. Circles represent observed individual results, a blue line represents statistical smoothing of the data, and the gray area represents 95% confidence intervals of the smooth.

Figure 2 illustrates the relationship between uncorrected QT and HR and between QTc and HR for three corrections: QTcF, QTcB, and QTcN (defined below) for the ECG samples collected before treatment. Without a correction, QT tended to decrease as HR increased. QTcF undercorrected this trend: it still tended to decrease with an increase in HR. QTcB overcorrected: it tended to increase with an increase in HR.

QTcN was a population-specific correction derived by estimating an optimal value for *r* using pretreatment observations. The estimated value was 0.42, which is between the values 0.33 and 0.5 defining QTcF and QTcB, respectively. As shown in Fig. 2, there was no remaining correlation between QTcN and HR. Therefore, QTcN was used as the primary correction method in results described later.

### Model development and final model.

**(i) Exposure-response model.** The effects of pretomanid, the bedaquiline M2 metabolite, and moxifloxacin were retained in the final model, which had the form

$$\Delta \text{QTc}=\text{intercept}+{\text{slope}}_{\text{pretomanid}}\times {\text{concentration}}_{\text{pretomanid}}+{\text{slope}}_{\text{moxifloxacin}}\times {\text{concentration}}_{\text{moxifloxacin}}+{\text{slope}}_{\text{M2}}\times {\text{concentration}}_{\text{M2}}+\varepsilon$$

where ε is the residual error.

Exploratory plots suggested that the bedaquiline M2 metabolite was the active component for QTc prolongation, consistent with prior knowledge (6); therefore, the plasma M2 concentrations were used in the modeling. With data from only one trial, the slope for linezolid was estimated imprecisely and was not significantly different from zero. An absence of an effect of linezolid on QT is consistent with prior knowledge (15), so it was not retained in the model. Somewhat surprisingly, pyrazinamide did show marginal evidence of an effect; however, the *P* value of 0.09 for QTcN did not meet the criterion of a *P* of <0.001 for retention in the model. QTc prolongation is not a recognized risk for pyrazinamide (16), and a previous study found no significant concentration-QTc relationship for pyrazinamide (14).

The intercept term represents the model’s prediction of ΔQTc at zero concentrations of all of pretomanid, moxifloxacin, and M2. This term was modeled as a combination of three components: (i) a nonparametric term that was allowed to vary freely across studies, visits within studies, and times postdose within visits; (ii) a term that depended linearly on a subject’s baseline QTc; and (iii) a random effect, η, for each subject:

$$\text{intercept}=\left(\text{nonparametric term}\right)+{\text{slope}}_{\text{baseline}}\left[{\text{QTc}}_{\text{baseline}}–\left({\text{median QTc}}_{\text{baseline}}\right)\right]+\eta$$

Estimates of the model’s parameters for the three QT corrections are provided in Table 1. Note that the parameter estimates are similar across the corrections. The nonparametric term is described in the next section.

**TABLE 1:** Summary of final model parameters

Parameter	QTcN estimate (90% CI)	QTcF estimate (90% CI)	QTcB estimate (90% CI)
Slope_pretomanid_ [ms/(μg/ml)]	1.61 (1.28, 1.94)	1.54 (1.21, 1.88)	1.53 (1.18, 1.88)
Slope_M2_ [ms/(μg/ml)]	19.3 (15.2, 23.3)	18.3 (14.1, 22.4)	19.6 (15.2, 24)
Slope_moxifloxacin_ [ms/(μg/ml)]	2.60 (1.66, 3.54)	2.47 (1.52, 3.41)	2.77 (1.77, 3.76)
Slope_baseline_^*a*^	–0.305 (–0.342, –0.268)	–0.362 (–0.397, –0.327)	–0.251 (–0.288, –0.214)
SD of the residual error, ε (ms)	10.2 (10.1, 10.4)	10.4 (10.2, 10.6)	10.8 (10.6, 11.1)
SD of the random effect of the intercept, η (ms)	9.58 (9.06, 10.1)	9.39 (8.88, 9.94)	10.5 (9.95, 11.1)

a The median baseline QTcN was 405 ms, that of QTcF was 390 ms, and that of QTcB was 415 ms.

**FIG 3:**
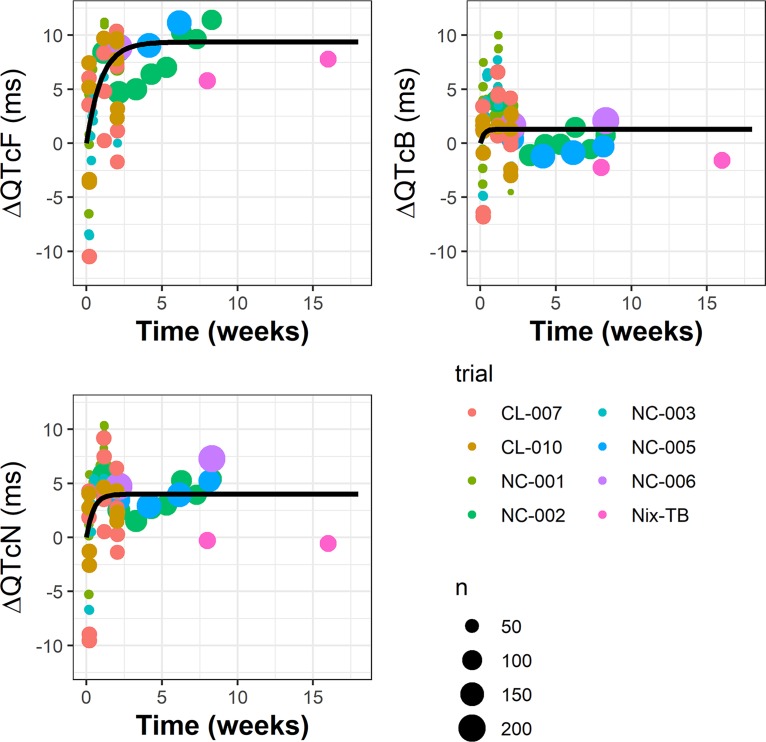
Estimated intercepts versus study week and the secular trend for QTcN, QTcF, and QTcB. Points represent the mean intercepts estimated for each study, visit, and time postdose at median baseline QTc, and the size of the points indicates the cohort size. Black curves represent the fitted secular trend model, *E_w_* = *P*_max_ (1 – *e*^–λ^*^w^*), where *P*_max_ is the steady-state value and log(2)/λ is the half-life for the approach to steady state.

**(ii) Secular trend.** The nonparametric intercept terms were examined further. Figure 3 shows that the estimated mean intercept (by study, visit, and time postdose for median baseline QTc) increased within the first few weeks and then plateaued. This long-term increase, termed the secular trend, was modeled as an asymptotic exponential function:

$$\text{nonparametric intercept term}=\text{secular trend}+\delta =P_{\text{max}}\times \left(1-e^{\mathrm{-\lambda}w}\right)+\delta$$

where *P*_max_ and λ are parameters, *w* is week of treatment, and δ is the residual error. Parameter estimates for this model are provided in Table 2. The secular trend model parameters were estimated using all the mean intercept terms (*Eo_jkt_*; see Materials and Methods) and weighted by sample size.

**TABLE 2:** Secular trend model parameters

Parameter	Value for:		
	QTcN	QTcF	QTcB
Pmax (ms)	3.99 (3.87–4.11)^*a*^	9.36 (9.13–9.59)	1.29 (1.19–1.38)
λ (1/wk)	2.3 (1.92–2.67)	1.03 (0.94–1.12)	4.64 (2.69–6.6)
SD of δ (ms)	3.19	4.47	2.87

a Values in parentheses are 95% CIs.

Thus, ignoring baseline effects and random errors, the modeled ΔQTc could be decomposed as follows: ΔQTc = (secular trend) + (concentration effects).

The studies included in this analysis had no placebo arms. The above decomposition is analogous to the decomposition applied in the case of a TQT study with placebo periods: ΔQTc = (placebo response) + (placebo-corrected change from baseline due to drug).

The second term above (i.e., placebo-corrected change from baseline due to drug) is conventionally denoted ΔΔQTc. We adapt that notational convention to the present circumstance and express ΔQTc as follows: ΔQTc = (secular trend) + ΔΔQTc.

That is, here ΔΔQTc refers to the following contribution:

$${\text{slope}}_{\text{pretomanid}}\times {\text{concentration}}_{\text{pretomanid}}+{\text{slope}}_{\text{moxifloxacin}}\times {\text{concentration}}_{\text{moxifloxacin}}+{\text{slope}}_{\text{M2}}\times {\text{concentration}}_{\text{M2}}$$

ΔΔQTc will be referred to as the secular-trend-corrected QTc change from baseline.

Interestingly, in Fig. 3, the observations from the BPaL regimen in the Nix-TB study, a focus of the analysis here, depart from the fitted trend lines, with intercepts closer to zero, although there are only two data points (only two postbaseline visits in the Nix-TB study with ECGs and concurrent PK), and the variability of all of the data about the trend is rather high. No explanation for this finding can be definitively offered, although it may be noted that the Nix-TB study was unique not just for its regimen, BPaL, but also for its patient population, XDR-TB and TI/NR MDR-TB patients.

### Model simulations.

**FIG 4:**
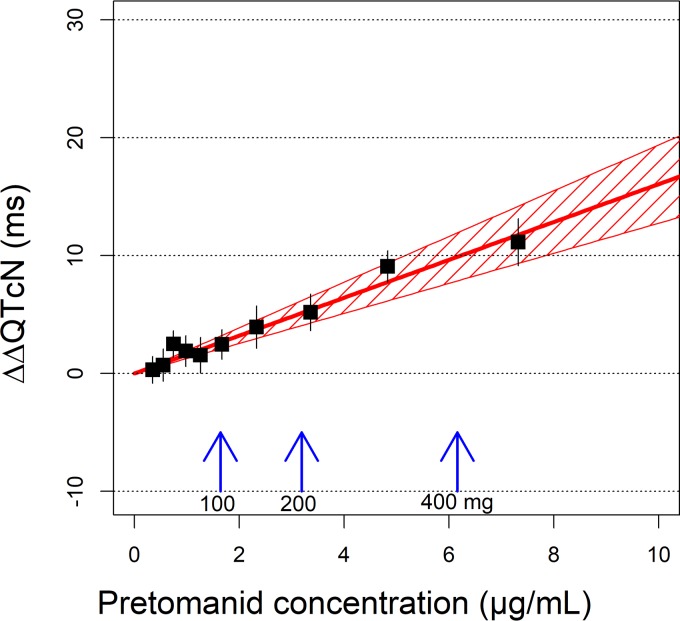
Exposure response relationship of ΔΔQTcN in subjects treated with pretomanid alone. The red line represents the model-predicted concentration ΔΔQTcN, and the shaded area indicates 90% confidence intervals (CIs). Squares represent the means of “observed” ΔΔQTcN values binned by plasma concentrations of pretomanid, and the error bars represent 90% CIs. The “observed” ΔΔQTcN was the measured ΔQTcN corrected with model-predicted intercept term. Blue vertical arrows indicate the typical maximum pretomanid concentrations at steady state for the pretomanid doses administered daily in the fed state.

**(i) Pretomanid alone.** Figure 4 shows the model-predicted, secular-trend-corrected QTcN change from baseline (ΔΔQTcN) as function of pretomanid plasma concentration. This represents the QTcN response to instantaneous pretomanid exposure. The mean and upper limit of the 90% confidence interval (CI) of ΔΔQTcN for various pretomanid doses are provided in Table 3. At each dose, the maximum pretomanid concentration at steady state (*C*_max,ss_) of a typical DS-TB subject in the fed state was determined using a population PK model reported elsewhere (unpublished data) and used for the simulation of ΔΔQTcN.

**TABLE 3:** Model-predicted ΔΔQTcN and ΔQTcN in subjects treated with pretomanid alone

Parameter	Value at indicated dose (mg)		
	100	200	400
*C*_max,ss_ (μg/ml)^*a*^	1.6	3.2	6.2
Concn-driven response			
ΔΔQTcN (ms) mean	2.6	5.1	9.9
ΔΔQTcN (ms) upper limit of 90% CI	3.2	6.2	12.0
Concn-driven response + maximum secular trend (4.0 ms)			
ΔQTcN (ms) mean	6.6	9.1	13.9
ΔQTcN (ms) upper limit of 90% CI	7.2	10.2	16.0

a Maximum pretomanid concentration at steady state of a typical DS-TB subject.

At a daily dose of 200 mg, the pretomanid *C*_max,ss_ was 3.2 μg/ml, resulting in a predicted ΔΔQTcN of 5.1 ms, with an upper limit of the 90% CI of 6.2 ms. At a daily dose of 100 mg, where the pretomanid *C*_max,ss_ was 1.6 μg/ml, the predicted mean and upper limit of the 90% CI of ΔΔQTcN were 2.6 and 3.2 ms, respectively.

Because of the secular trend, the actual QTc change from baseline without correction, i.e., ΔQTcN, would be higher than the effect driven by pretomanid concentration alone. The mean ΔQTcN was predicted to be 9.1 ms, with an upper limit of the 90% CI of 10.2 ms, for dosing pretomanid 200 mg QD until the secular trend reaches its maximal, asymptotic value of 4.0 ms.

**FIG 5:**
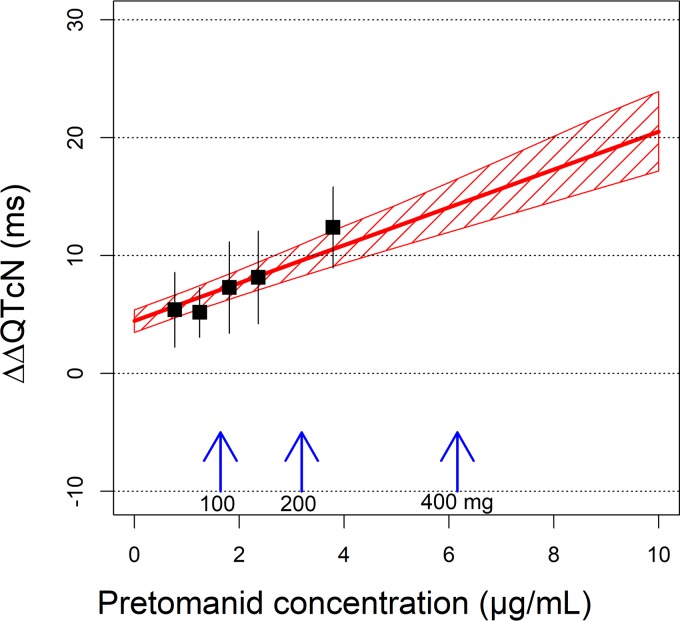
Exposure response relationship of ΔΔQTcN in subjects treated with the BPaL regimen. The red line represents the model-predicted mean values of ΔΔQTcN versus pretomanid concentrations, and the shaded area indicates 90% confidence intervals (CIs). Squares represent the means of “observed” ΔΔQTcN values binned by plasma concentrations of pretomanid, and the error bars represent 90% CIs. The “observed” ΔΔQTcN was the measured ΔQTcN corrected with model-predicted intercept term. Blue vertical arrows indicate typical maximum pretomanid concentrations at steady state for the pretomanid doses administered daily in the fed state. The mean bedaquiline M2 concentration of 0.25 μg/ml observed in the Nix-TB study was used in the simulation.

**(ii) Pretomanid in the BPaL regimen.** For the BPaL regimen used in the Nix-TB study, the active QTc components were pretomanid and the bedaquiline M2 metabolite. The relationship between ΔΔQTcN and pretomanid concentrations is shown in Fig. 5 (where the zero concentration of pretomanid should be interpreted as the impact of the bedaquiline M2 metabolite alone). In the Nix-TB study, the mean bedaquiline M2 concentration of 0.25 μg/ml was estimated to induce a mean QTcN increase of 4.5 ms. Adding pretomanid further increased the ΔΔQTcN. For the 200-mg regimen, the mean steady-state ΔΔQTcN was predicted to be 9.6 ms, with an upper 90% CI limit of 11.0 ms (Table 4).

**TABLE 4:** Model-predicted ΔΔQTcN and ΔQTcN in subjects treated with the BPaL regimen^*a*^

Parameter	Value at indicated dose (mg)			
	0	100	200	400
*C*_max,ss_ (μg/ml)^*b*^	0	1.6	3.2	6.2
Concn-driven response				
ΔΔQTcN (ms) mean	4.5	7.1	9.6	14.4
ΔΔQTcN (ms) upper limit of 90% CI	5.4	8.2	11.0	16.5
Concn-driven response + maximum secular trend (4.0 ms)				
ΔQTcN (ms) mean	8.5	11.1	13.6	18.4
ΔQTcN (ms) upper limit of 90% CI	9.4	12.2	15.0	20.5

a The mean bedaquiline M2 concentration of 0.25 μg/ml observed in the Nix-TB study was used in the simulation.

b Maximum pretomanid concentration at steady state of a typical DS-TB subject.

Including the secular trend estimated from all the trials, ΔQTcN values for the BPaL regimen were predicted to be 13.6 and 15.0 ms for the mean and upper limit of the 90% CI, respectively. However, it may be noted from Fig. 3 that the estimated intercepts in the Nix-TB study tended to be lower than those in the other trials, approximately zero for QTcN. Using zero for the secular trend would, by definition, render the predicted ΔQTcN the same as the ΔΔQTcN.

## DISCUSSION

The time-matched plasma concentration and QT results in subjects with TB from eight clinical studies in the development program of pretomanid were evaluated in this analysis. The impact of pretomanid alone, as well as in various combinations with other TB drugs, including bedaquiline, moxifloxacin, pyrazinamide and linezolid, was evaluated.

The recommended clinical dose of pretomanid is 200 mg QD. At that dose, the typical steady-state maximum concentration among DS-TB subjects dosed in the fed state is 3.2 μg/ml. At that concentration, the model predicted a mean ΔΔQTcN of 5.1 ms and an upper limit of the 90% CI of 6.2 ms, well below the 10-ms threshold of regulatory concern for the CI upper limit (10). The corresponding mean and upper limit for ΔQTcN were 9.1 ms and 10.2 ms at steady state.

At a lower dose of 100 mg, the typical steady-state maximum concentration among DS-TB subjects dosed in the fed state is 1.6 μg/ml. At that concentration, the model predicted a mean ΔΔQTcN of 2.6 ms and an upper limit of the 90% CI of 3.2 ms.

These results are in line with those of a TQT study (ClinicalTrials.gov Identifier NCT01674218; unpublished data), which was performed in healthy volunteers and did not contribute to the analysis here. In that study, pretomanid was administered as a single dose in the fasted state, and the geometric mean *C*_max_ values of pretomanid were 1.27 μg/ml and 2.33 μg/ml for doses of 400 mg and 1,000 mg, respectively. The maximum least-squares-mean ΔΔQTcI values were 2.7 ms and 4.4 ms for the 400-mg and 1,000-mg doses, respectively, and the upper limits of the 90% CIs did not exceed 4.4 ms and 6.1 ms, respectively.

Table 5 summarizes these results about pretomanid alone from the concentration/QTc model and the TQT study.

**TABLE 5:** ΔΔQTc for pretomanid alone from the concentration-QTc model and the thorough QT study

Pretomanid concn (μg/ml)^*a*^	Source	ΔΔQTc^*b*^ (ms) (estimate^*c*^ [90% CI upper limit])^*d*^
1.27	Thorough QT study	2.7 (≤4.4)
1.64	Concentration-QTc model	2.6 (3.2)
2.33	Thorough QT study	4.4 (≤6.1)
3.19	Concentration-QTc model	5.1 (6.2)

a In the thorough QT study, values are the geometric mean *C*_max_ after a single dose. In the modeling analysis, values are median steady-state *C*_max_.

b For the thorough QT study, the value given is the ΔΔQTcI, the placebo-adjusted change from baseline in the individual correction of QTc. For the modeling analysis, the value given is the ΔΔQTcN, the secular-trend-adjusted change from baseline for the population-specific correction of QTc.

c For the thorough QT study, the estimate is the maximum least-squares-mean value. For the modeling analysis, the estimate is the mean based on simulations of the model.

d For the thorough QT study, the 90% CI upper limit is based on all confidence intervals at QT observation times postdose. For the modeling analysis, the confidence limit is based on simulations of the model.

Thus, the pretomanid concentration/QTc response was consistent across studies and subjects with or without TB. Also, the concentration/QTc response was consistent for QTcF, QTcB, and QTcN.

In addition to pretomanid alone, another focus of this work was the BPaL regimen used in the Nix-TB study. Bedaquiline was found to affect QTc via its M2 metabolite. Linezolid was found not to have a significant effect. At a daily dose of 200 mg of pretomanid, the BPaL regimen tested in the Nix-TB study was predicted to have a mean ΔΔQTcN of 9.6 ms, with an upper limit of the 90% CI of 11.0 ms. The corresponding mean and upper limit for ΔQTcN were 13.6 and 15.0 ms at steady state.

Moxifloxacin was also found to significantly affect QTc. A phase 2c study, SimpliciTB (ClinicalTrials.gov Identifier NCT03338621), is under way to evaluate the safety and efficacy of a regimen of bedaquiline, pretomanid, moxifloxacin, and pyrazinamide. Further quantitative assessment of that regimen awaits the results of that study.

Increased heart rate (HR) in subjects infected with tuberculosis has been previously documented (13). Decreases of HR on treatment, by 5 to 10 bpm, were observed in the clinical studies. A potential explanation is that subjects’ HRs normalized once the disease was under control. To account for the difference in QT at different HRs, the analysis examined various correction methods. A negative slope was found in the plot of QTcF versus HR; therefore, a decrease in HR after treatment would contribute to an increase in QTcF. In contrast, QTcB had a positive slope with HR, and a decrease of HR would lead to a decrease of QTcB. The QTcN correction was not correlated with HR, so the QTcN results would be expected to be less biased by changes in HR on treatment.

The selection here of QTcN for primary analysis was based on pretreatment evaluations. There were too few such pretreatment observations for analyses based on individual QT-versus-HR relationships (17). Others have included on-treatment comparisons of correction factors in their choice (14). That was not done here for fear that incorporating on-treatment data into the definition of the response variable might bias the assessed concentration-response relationship. On the other hand, some researchers have argued that on-treatment values of HR should be included as a covariate in a model for uncorrected QT (18). If the log-transformed scale is used, such a model would make estimation of the population-specific correction factor, *r*, part of modeling the on-treatment data; and an intersubject random effect on the RR term would even allow individual correction, albeit again determined by the on-treatment data. This approach has not yet been encouraged by regulators (9, 10), but it suggests that an optimal solution may not yet have been found for the difficulty in interpreting QT data when QT and HR change in response to long-term treatment, as in antituberculosis therapy.

A solution to that complexity that was adopted here was to separate the time-dependent effect of treatment from the concentration-dependent effect. The time-dependent effect was incorporated into the intercept of the typically assumed (9) linear relationship between plasma drug concentration and change from baseline in QTc. A linear model was fitted to each study-by-visit-by-time-postdose grouping of data, with the coefficients (slopes) of the concentration dependence remaining constant across those groupings but the intercepts being estimated separately for each such grouping. Then, in a second step, the pattern of those intercepts’ variation with time on treatment was modeled. That pattern was termed the secular trend.

Depending on the QT correction method, the secular trend could take several weeks to reach steady state. The maximum values of the secular trend were 9.4, 1.3, and 4.0 ms for QTcF, QTcB, and QTcN, respectively. One major source of the secular trend appears to be the decrease of HR. An increase in the estimated intercept term was correlated with a decrease in heart rate (see Fig. S1 in the supplemental material). The secular trend estimated using QTcF would thus be overestimated because of the negative QTc-versus-HR relationship. The secular trend estimated in QTcB, on the other hand, would be underestimated due to its positive QTc-versus-HR relationship. Therefore, the secular trend estimated for QTcN was considered to be the least biased. Although the exact cause of the secular trend is unknown, possible reasons besides HR decrease include accumulation of other metabolites, myocardial accumulation of the drug/metabolites, and other long-term physiological/biological changes as a result of treatment. Of note, between-study variability of the intercept was relatively high.

Additional limitations of the present work involve features of concentration/QTc modeling that are sometimes found important and useful but were not considered because of the sparseness of the available data, namely, hysteresis and circadian variation.

Finally, it should be noted that whereas QT remains an important outcome of scientific and regulatory interest, its primary value is as a biomarker for the risk of arrhythmias such as Torsade des Pointes. In that regard, it is an imperfect biomarker. The underlying electrophysiology involves a complex interplay of several different types of ion channels. Also, many other intrinsic and extrinsic factors are known to affect the relationship between QT prolongation and the occurrence of arrhythmias (5).

## MATERIALS AND METHODS

The analysis followed the ICH guideline for concentration-QT analysis (10). The scientific white paper on concentration-QTc modeling by (9) was used as a reference for model building.

### Data.

Data included in the concentration-QTc analysis came from eight clinical studies: CL-007 (19), CL-010 (20), NC-001 (21), NC-002 (22), NC-003 (23), NC-005 (3), STAND (ClinicalTrials.gov Identifier NCT02342886), and Nix-TB (4). Treatment regimens in NC-003 that included clofazimine were excluded, as were subjects from control arms of HRZE (isoniazid, rifampin, pyrazinamide, ethambutol) in all studies.

ECGs were evaluated using a 12-lead ECG method. Singular or triplicate ECG results were collected before and after the start of treatments. Drug plasma concentrations, including those for pretomanid, bedaquiline, bedaquiline’s main metabolite (M2), moxifloxacin, pyrazinamide, and linezolid, were obtained for measurement at the same time as the ECG collection. Only ECGs time matched with valid drug concentrations were used in the modeling analyses.

Mean ECG results of the triplicates were used in the analysis. Baseline ECG parameters were calculated using the average results collected at screening, on the day before the start of treatment, and predose on the first day of treatment. For the majority of the subjects (76% of the total population), the baseline was calculated using two ECG samples, one collected at the day before the treatment and the other at predose on the first day of treatment. Study NC-005 (20% of the total population) used only one sample collected at day 1 predose, and study NC-003 (4% of the total population) used five ECG samples collected before treatment.

### QT correction methods.

Besides Fridericia’s (11) correction (QTcF) and Bazett’s (12) correction (QTcB), a population-specific correction factor method (QTcN) was also used for the analysis. The correction formula is given as follows: QTcN = QT/*RR^r^*, where *RR* = 60/HR and the correction factor, *r*, is the linear regression slope of the log(QT) ∼ log(*RR*) relationship derived using the pretreatment QT results. When *r* is 1/3, the corrected QT becomes QTcF, and when *r* is 1/2, it becomes QTcB.

The same correction factor was used for all the subjects for QTcN. The individual correction method (QTcI) was not used in this analysis due to the limited number of pretreatment observations per subject. Except for the subjects in the NC-003 study, subjects had only one or two pretreatment QT observations.

**(i) Model development.** The change from baseline of QTc (ΔQTc) for the *i*th subject at time post dose *t* of the *k*th visit of the *j*th study was analyzed by the following general models:

$$\begin{array}{l} \Delta \mathrm{QT}c_{\mathrm{ijkt}}=Eo_{\mathrm{ijkt}}+f\left(\theta ,C_{\mathrm{ijkt}}\right)+\varepsilon_{\mathrm{ijkt}}\\ Eo_{\mathrm{ijkt}}=Eo_{\mathrm{jkt}}+e_{o}I\times \left(\mathrm{BQT}c_{i}-\text{median}\mathrm{BQTc}\right)+\eta_{i}\\ f\left(\theta ,C_{\mathrm{ijkt}}\right)=\mathrm{sl}\times C{\left(t\right)}_{\mathrm{ijk}}\\ \varepsilon_{\mathrm{ijkt}}∼N\left(0,\sigma^{2}\right)\\ \eta_{i}∼N\left(0,\omega^{2}\right) \end{array}$$

In these equations, ΔQTc*_ijkt_* is the change from baseline in QTc for the *i*th subject at time postdose *t* of the *k*th visit of the *j*th study. *Eo_ijkt_* is the intercept. To account for heterogeneity among studies, visits, and times postdose, a different mean intercept, *Eo_jkt_*, was estimated for each time postdose of each visit of each study; i.e., a nonparametric model was used for the intercepts. η*_i_* is a between-subject random effect, assumed to be normally distributed with mean 0 and variance ω^2^. Different ω values by study and by number of ECG samples used for calculating the baseline were evaluated but found not to be needed. *f*(θ,*C_ijkt_*) is the relationship between drug concentrations, *C*(*t*), and ΔQTc with the slope of sl. A nonlinear concentration response model (i.e., Emax model) was also evaluated, although it was not retained in this analysis. ε*_ijkt_* is the residual error with mean 0 and variance σ^2^. Values of σ^2^ were allowed to differ between single ECGs versus means of triplicates but found not to be needed. The impact of the baseline QTc on the intercept was estimated by including the baseline QTc (BQTc), centered at its median value, as a covariate on the intercept with coefficient *e_o_I*. The same model structures were applied to pretomanid, bedaquiline M2, and moxifloxacin concentrations.

The combined ΔQTc effect of two or more drugs was modeled as additive of the individual effects plus interactions between drugs. A three-drug combination was modeled as:

$$f\left(\theta ,C_{\mathrm{ijkt},\text{drug}1},C_{\mathrm{ijkt},\text{drug}2},C_{\mathrm{ijkt},\text{drug}3}\right)=f_{\text{drug}1}+f_{\text{drug}2}+f_{\text{drug}3}+r_{12}\left(f_{\text{drug}1}\cdot f_{\text{drug}2}\right)+r_{13}\left(f_{\text{drug}1}\cdot f_{\text{drug}3}\right)+r_{23}\left(f_{\text{drug}2}\cdot f_{\text{drug}3}\right)$$

where *f*_drug 1_ is the individual effect of drug 1 given alone, etc., and *r*_12_ is the interaction coefficient between drugs 1 and 2, etc. The interaction terms were originally included during the model development. However, they were not estimated precisely and were not significantly different from zero (*P* = 0.614), Thus, they were removed from the final model.

An initial model was developed using data from subjects dosed with pretomanid alone. The model was subsequently expanded by including subjects dosed with other drugs and combinations. Significant model parameters were retained in the model based on the likelihood ratio test of nested models (with *P* < 0.001). Only the drugs with significant concentration-QTc effects were included in the final model.

Model development was conducted separately using the QTcN and the QTcF data. Both developments resulted in the same final model, which was then also applied to QTcB.

### Covariates.

The impacts of TB type (DS-TB, MDR-TB, or XDR-TB) and baseline QTc on the intercept of the concentration-QTc relationship were evaluated during the model development. The study (CL-007 versus CL-010) was tested as a covariate on the pretomanid-concentration slope in the pretomanid-alone model. Significant covariates were retained in the model, based on the likelihood ratio test (*P* < 0.001).

### Secular trend.

The intercepts of the linear relationships between ΔQTc and drug concentrations, estimated for each time postdose at each visit of each study, were plotted versus time. A pattern was found, which was called the secular trend. Unlike the concentration-QTc response, the secular trend was not immediately related to the drug concentration but rather was a reflection of factors that may have had long-term effects on the QT (e.g., change of heart rate, myocardial accumulation of drug, etc.). An equation was used to describe the secular trend over time, given as follows:

$$Eo_{\mathrm{jkt}}=E_{w\left(\mathrm{jk}\right)}+\delta_{\mathrm{jkt}}$$

$$E_{w}=P_{\text{max}}\times \left(1-e^{-\lambda w}\right)$$

where *w*(*jk*) is the week of the *k*th visit in the *j*th study, *E_w_* is the secular trend at week *w*, *P*_max_ is the maximum value of *E_w_* at steady state, λ is the onset slope such that log(2)/λ is the half-life for the time to achieve steady state, and δ*_jkt_* is a residual error with mean 0.

### Simulations.

Simulations of the model were performed as follows. Let $\hat{sl_{\text{Pa}}}$ and slM2^ be the estimated slopes for pretomanid (Pa) and M2. Let sePa^ be the estimated standard error of $\hat{sl_{\text{Pa}}}$, and let $\hat{{\text{Cov}}_{\text{Pa, M2}}}$ be the estimated covariance matrix of ($\hat{sl_{\text{Pa}}}$, $\hat{sl_{\text{M2}}})$. Let *C*_Pa_ denote a concentration of pretomanid.

For pretomanid alone, the mean and confidence bounds of ΔΔQTcN were computed as $\hat{sl_{\text{Pa}}}\times C_{\text{Pa}}$ and $\left(\hat{sl_{\text{Pa}}}\pm 1.645\times \hat{se_{\text{Pa}}}\right)\times C_{\text{Pa}}$.

For BPaL, 1,000 values of (*sl*_Pa_, *sl*_M2_) were sampled from a bivariate normal distribution with mean ($\hat{sl_{\text{Pa}}}$, $\hat{sl_{\text{M2}}}$) and covariance matrix $\hat{{\text{Cov}}_{\text{Pa, M2}}}$. Means and confidence limits of ΔΔQTcN were derived from the 1,000 resulting values of *sl*_Pa_ × *C*_Pa_ + *sl*_M2_ × 0.25. Note that the average M2 concentration from the Nix-TB study, 0.25 μg/ml, was assumed.

Means and confidence limits for ΔQTcN were computed by adding the estimated asymptotic value of the secular trend, $\hat{P_{\text{max}}}$, to the corresponding values for ΔΔQTcN; thus, uncertainty in $\hat{P_{\text{max}}}$ was not incorporated.

### Software.

The analyses were performed using R v3.3.2 in addition to CRAN packages (24). Mixed-effect modeling was performed using the nlme package for R (25).

## Supplementary Material

Supplemental file 1
