# Supplementary material for: Long-Term Effects on QT Prolongation of Pretomanid Alone and in Combinations in Patients with Tuberculosis
Source: Antimicrob Agents Chemother. 2019 Sep 23;63(10):e00445-19. doi: 10.1128/AAC.00445-19 (PMC6761551; doi:10.1128/AAC.00445-19)
Supplement: Supplemental file 1 [file AAC.00445-19-s0001.pdf]

SUPPLEMENTARY MATERIAL

Table S1. Overview of ECG data and baseline characteristics by study

Study	N subject	N ECG (post-dose with PK)	QTcF baseline (ms)	Heart rate baseline (bpm)	Age (yr)	Weight (kg)	Sex (F/M)	Race (A/B/M/N/O/W)	TB type (DS/MDR/XDR)
CL-007	61	826	392.2 (346.7 to 428.8)	88.5 (58.7 to 124.3)	30 (18 to 56)	53 (39 to 72.5)	61/0	0/32/29/0/0/0	61/0/0
CL-010	61	595	384 (348.5 to 426.8)	90.3 (49.5 to 133.5)	26 (18 to 55)	52 (40 to 73)	61/0	0/24/37/0/0/0	61/0/0
NC-001	75	741	395 (359.2 to 433)	84.3 (55 to 114)	27 (19 to 53)	53.8 (41.4 to 79.4)	18/57	0/29/46/0/0/0	75/0/0
NC-002	139	949	385.5 (337.5 to 427.5)	90 (54.5 to 134.5)	27 (18 to 65)	55 (40 to 90.6)	49/90	0/97/41/0/1/0	116/23/0
NC-003	30	416	387.6 (345.2 to 417.8)	92.4 (59.9 to 117.3)	29.5 (19 to 62)	49.4 (40.4 to 68.1)	21-Sep	1/13/0/0/15/1	30/0/0
NC-005	166	584	388.8 (317 to 446)	88.5 (51.3 to 158.7)	32 (18 to 69)	53 (35 to 99.8)	40/126	0/138/26/1/0/1	111/55/0
NC-006	214	400	392.5 (348.5 to 460.5)	88 (54.5 to 138)	35.5 (18 to 77)	52.2 (31.8 to 137.2)	66/148	15/146/31/0/12/10	202/12/0
Nix-TB	84	141	415.2 (368.5 to 469)	84.2 (50 to 131.5)	33.5 (18 to 60)	54.5 (30 to 108)	41/43	0/64/0/0/19/1	0/33/51
Total	830	4652	391.5 (317 to 469)	88.5 (49.5 to 158.7)	31 (18 to 77)	53 (30 to 137.2)	345/485	16/543/210/1/47/13	656/123/51

Race: A-Asian, B-black, M-mixed, N-native Hawaiian or Pacific islander, O-other and W-white. Continuous variables are report as median(range).

Table S2. Summary of analysis dataset by treatment

Drugs	N trial	N Arm	N Subj.	N QT obs	Dose Pretomanid (mg)	Dose Bedaquiline (mg)	Dose Moxifloxacin (mg)	Dose Pyrazinamide (mg)*	Dose Linezolid (mg/day)	Base QTcF (ms)	ΔQTcF (ms)
pa	2	8	122	1421	50/100/150/ 200/600/1000/1200	0	0	0	0	387.7 (346.7 to 428.8)	7.3 (-79.5 to 81)
bed	1	1	15	100	0	700	0	0	0	389 (365 to 425.5)	5.5 (-19 to 50)
pyr	1	1	15	209	0	0	0	1500	0	389.6 (345.2 to 417.8)	-2.3 (-40.3 to 48.6)
pa+bed	1	1	15	28	200	700	0	0	0	400.5 (364.5 to 426.5)	18.8 (1.5 to 45)
pa+pyr	1	1	15	258	200	0	0	1500	0	390 (365.4 to 419)	8 (-30.3 to 49.3)
bed+pyr	1	1	15	102	0	700	0	1500	0	399.5 (367 to 433)	9 (-34.5 to 36.5)
pa+mox+pyr	3	8	368	1602	100/200	0	400	1500	0	387 (337.5 to 460.5)	13.8 (-38 to 81.5)
pa+bed+pyr	2	3	126	591	200	200/400	0	1500	0	387.3 (317 to 446)	17.1 (-31.3 to 125)
pa+bed+lin	1	1	84	141	200	200	0	0	600	418 (368.5 to 469)	6 (-45 to 44.5)
pa+mox+bed+pyr	1	1	55	200	200	200	400	1500	0	392.3 (333.7 to 431.3)	18.5 (-13.3 to 86.7)
Total	8	26	830	4652	0/50/100/150/ 200/600/1000/1200	0/200/400/700	0/400	0/1500	0/600	389 (317 to 469)	11 (-79.5 to 125)

Pa- Pretomanid (PA-824), bed-bedaquiline, mox-moxifloxacin, pyr- pyrazinamide, Lin – Linezolid. *some trials were dosed by weight. [@]As 600 mg BID or 1200 mg QD; dose adjustments for toxicity were frequent. QTc variables are reported as median(range).

Figure S1. Intercept terms versus change of heart rate

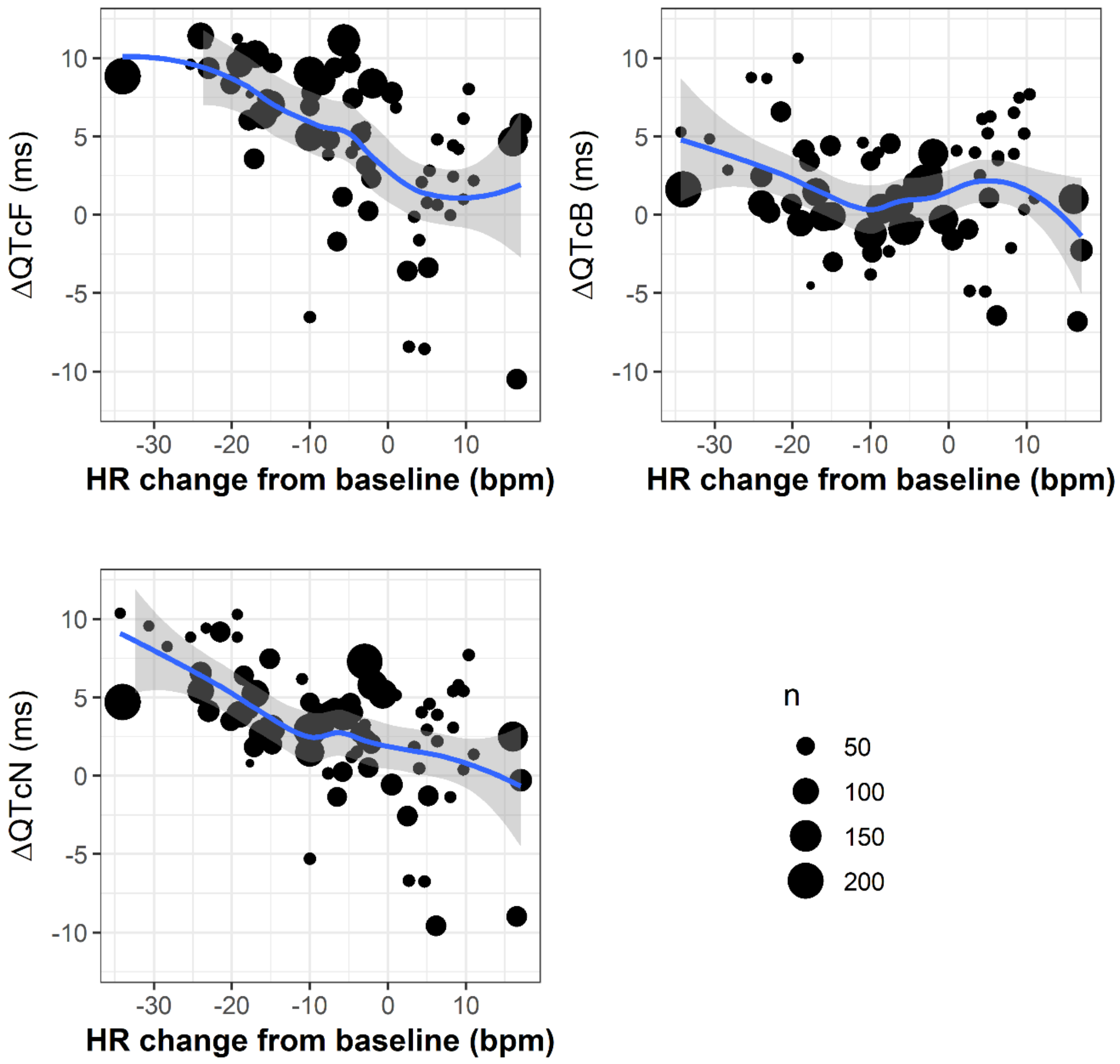

Dots are intercept terms estimated for each trial, visit and timepoint. The blue line is a statistical smooth of the data, the gray area represents confidence intervals of the smooth.
